# Social physique anxiety and physical activity

**DOI:** 10.1192/j.eurpsy.2021.2011

**Published:** 2021-08-13

**Authors:** A. Zartaloudi, D. Christopoulos

**Affiliations:** 1 Nursing, University of West Attica, Athens, Greece; 2 Nursing, Psychiatric Hospital of Athens “Dafni”, Athens, Greece

**Keywords:** Social Physique Anxiety, Physical Activity, athletics, motivation

## Abstract

**Introduction:**

Social Physique Anxiety is defined as an emotional response that reflects individuals’ concerns regarding the way their body may be observed or judged by others.

**Objectives:**

To explore the relationship between physical activity and social physique anxiety.

**Methods:**

A literature review haw been made through pubmed database.

**Results:**

Social Physique Anxiety is negatively related to participation in physical activity and commitment to exercise. Studies examining the relationship between motivation and social physique anxiety have shown that external motivations, such as improving muscle tone and body attractiveness, are directly linked to social physique anxiety. In addition, social physique anxiety is negatively related to self-efficacy. Individuals who believe that they will be judged by others to be ineffective are less likely to be engaged in physical activity programs. Social Physique Anxiety has been linked to negative effects on mental health such as low self-esteem, smoking and eating disorders.

**Conclusions:**

Given all the negative effects of social physique anxiety, as it is responsible for a wide range of health-related behaviors, it is important to identify physical activity-related motivational mechanisms in order to reduce the impact of social physique anxiety.

**Disclosure:**

No significant relationships.

